# Supplementary figures and images for: Total Phenolic Fraction (TPF) from Extra Virgin Olive Oil: Induction of apoptotic-like cell death in Leishmania spp. promastigotes and in vivo potential of therapeutic immunomodulation
Source: PLoS Negl Trop Dis. 2021 Jan 11;15(1):e0008968. doi: 10.1371/journal.pntd.0008968 (PMC7799795; doi:10.1371/journal.pntd.0008968)

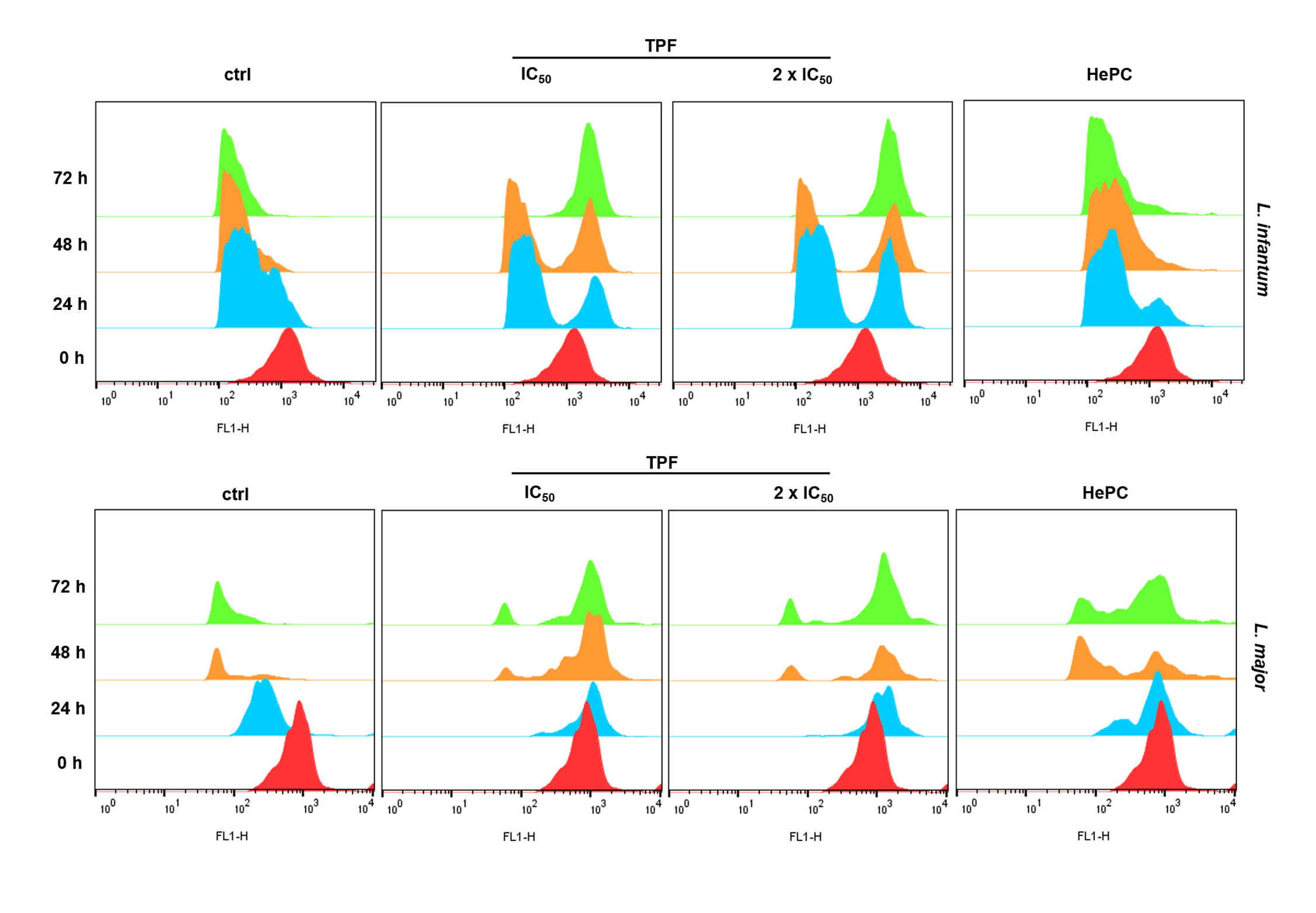

Supplement: S1 Fig — L. infantum and L. major early exponential-phase promastigotes were treated with IC50 and 2 x IC50 concentrations of TPF and their proliferation rate was qualitatively monitored at 24 h intervals for 3 consecutive days by CFSE staining and subsequent analysis of fluorescence intensity in FACS. HePC (IC50)-treated and untreated parasites were used as positive and negative control groups, respectively. The results are presented as single parameter histogram overlays representative of one experiment. (TIF) [file pntd.0008968.s001.tif]

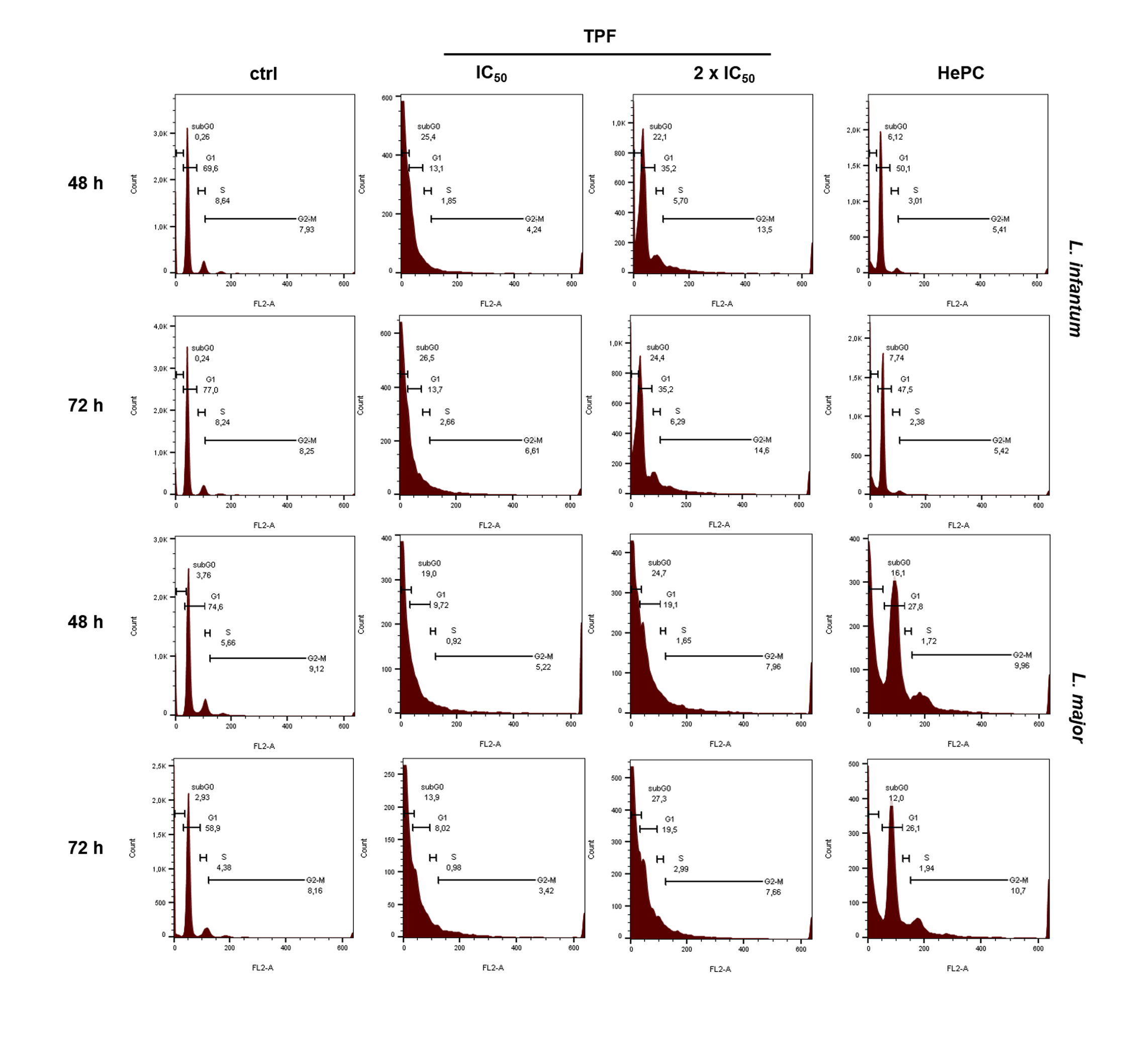

Supplement: S2 Fig — Exponential-phase L. infantum and L. major promastigotes were either left untreated or were treated with IC50 and 2 x IC50 concentrations of TPF and HePC (IC50) for 48 and 72 h. Parasite cell cycle was analyzed through FACS and the results are plotted as single parameter histograms representative of one experiment. (TIF) [file pntd.0008968.s002.tif]

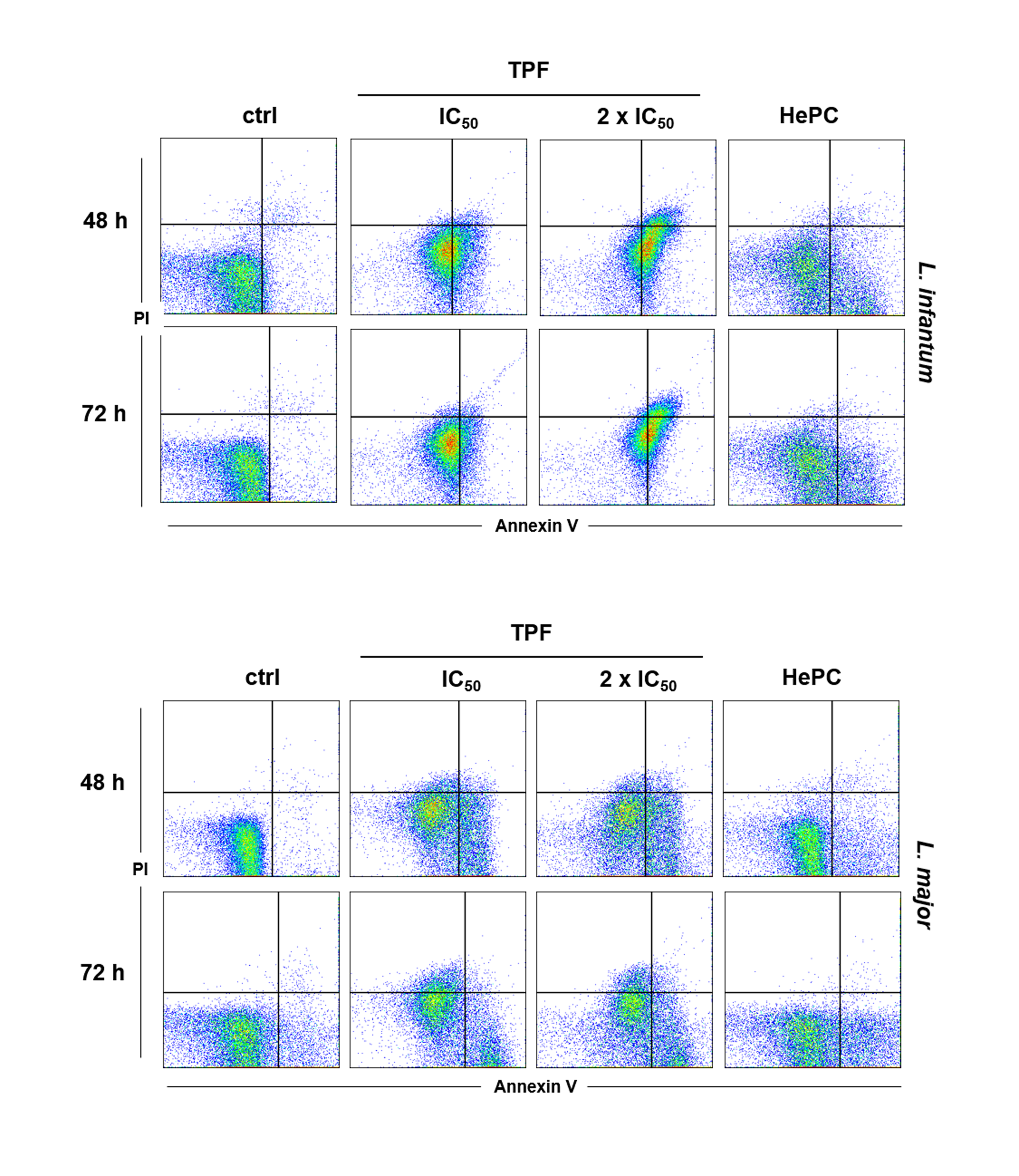

Supplement: S3 Fig — Exponential-phase L. infantum and L. major promastigotes were either left untreated (negative control) or were treated with IC50 and 2 x IC50 concentrations of TPF and HePC (IC50, positive control) for 48 and 72 h. At the end of the aforementioned time-points, parasites were double stained with annexin V-FITC and PI and were analyzed by FACS. The results are presented as flow cytometric dot plots with respective quadrants, representative of one experiment. (TIF) [file pntd.0008968.s003.tif]
