# Supplementary material for: Total Phenolic Fraction (TPF) from Extra Virgin Olive Oil: Induction of apoptotic-like cell death in Leishmania spp. promastigotes and in vivo potential of therapeutic immunomodulation
Source: PLoS Negl Trop Dis. 2021 Jan 11;15(1):e0008968. doi: 10.1371/journal.pntd.0008968 (PMC7799795; doi:10.1371/journal.pntd.0008968)
Supplement: Attachment — Submitted filename: RESPONSE TO REVIEWERS.pdf [file pntd.0008968.s004.pdf]

RESPONSE TO REVIEWERS

Reviewer No. 1

Comments for the author

General Comment: "*It is a very poorly executed study, the data is represented very poorly and cannot be considered for any scientific journal. The starting sentence of 'Leishmaniasis being a disease unreliable chemotherapy' is an incorrect statement*."

Answer: Despite the disappointing reviewer's general comment, the manuscript has been almost re-written, necessary experiments were repeated or performed and data have been re-analyzed where it was suggested. We accept that the starting sentence of "Leishmaniasis is a serious multifactorial parasitic disease with unreliable current chemotherapy" was an incorrect statement and it has been replaced with "Leishmaniasis is a serious multifactorial parasitic disease with limited treatment options", in the revised manuscript.

Comment 1: *"The assessment of promastigote growth by differential counting of dead and live promastigotes by using the Trypan blue exclusion dye is a very crude and inaccurate approach. In view of the CFSE staining method available, this Trypan blue approach is redundant."*

Answer 1: The assessment of promastigote growth using the Trypan blue exclusion dye is indeed a crude approach and can be considered as redundant in the view of the CFSE staining method. Nevertheless, these methods for quantification of viable parasites using Trypan blue and the CFSE as a cell division marker, have been previously published in similar studies at yours and other scientific journals:

- *Christine Achiaa Antwi et al., In vitro activity and mode of action of phenolic compounds on Leishmania donovani, PLOS Neglected Tropical Diseases, 2019, DOI: 10.1371/journal.pntd.0007206*
- *Cynthia Mmalebna Amisigo et al., In vitro anti-trypanosomal effects of selected phenolic acids on Trypanosoma brucei, PlosOne, 2019,* DOI: 10.1371/journal.pone.0216078
- *I. Messaritakis et al., Leishmania donovani s.l.: Evaluation of the proliferation potential of promastigotes using CFSE staining and flow cytometry, Experimental Parasitology 125,2010, 384–388, DOI: 10.1016/j.exppara.2010.03.006*
- *Sarah W. Kamau et al., Flow cytometry analysis of the effect of allopurinol and the dinitroaniline compound (Chloralin) on the viability and proliferation of Leishmania infantum promastigotes, BMC Pharmacology, 2001, 1:1*, DOI: 10.1186/1471-2210-1-1

Moreover, the Trypan blue approach is considered as a quantitative approach while the CFSE assay as a more qualitative approach on parasite replication. Taking into consideration the

reviewer’s comment, in the revised manuscript, we answered the question of “what is the effect of TPF on parasite growth” by combining three methods. We firstly determined the effect of TPF on promastigote viability with the use of a resazurin-based assay and determined the effect of TPF on the proliferation rate of promastigotes using both Trypan blue approach and CFSE staining.

Comment 2: *“Line 210: Please state the incubation periods”.*

Answer 2: The incubation periods are now mentioned in lines 241 – 242 of the revised manuscript. The relevant incubation periods have also being clarified in line 256 and lines 274 - 275 of the revised manuscript.

Comment 3: *“A major problem in all assays is that it is stated that the concentration of the extract selected for this study is the IC50 and 2X1C50, and therefore the results section should begin with a viability assay that calculates this value. Nowhere could I find a numerical value for the IC50 which is a major drawback.”*

Answer 3: We accept the reviewer’s comment 3 that the numerical values for the inhibitory concentration were not obvious. To this purpose, we added Table 1 with the half-maximal inhibitory concentrations (IC_{50} values) of TPF against *L. infantum* and *L. major* promastigotes, in the revised manuscript. Moreover, we added one paragraph in Materials and Methods section describing the procedure for the determination of the IC_{50} values against *Leishmania* spp. promastigotes. Additionally, in Results section, we inserted one paragraph presenting the inhibitory effect of TPF on *L. infantum* and *L. major* promastigotes with the concomitant insertion of the relevant graph (Figure 1 in the revised manuscript).

Comment 4: “*How was the cell size determined by Flow Cytometry? A reference is needed of this methodology. Composite results is not provided, only a representative profile*.”

Answer 4: The relevant reference that is indeed needed to be added for the substantiated use of the methodology is:

- *Britta E.A. et al. Cell death and ultrastructural alterations in Leishmania amazonensis caused by new compound 4-Nitrobenzaldehyde thiosemicarbazone derived from S-limonene. BMC Microbiology 2014, 14:236, DOI: 10.1186/s12866-014-0236-0*

Nevertheless, we considered that the cell volume analysis of untreated and treated parasites by flow cytometry, is redundant in the view of the obtained microscopy images. For this reason, we withdrew the relevant methodology and figure, in the revised manuscript.

Comment 5: *"In Fig. 2A and 2B, why is there no cell growth, cells appear to be in stationary phase. However, all experiments pertaining to cell growth should always be performed with log phase cells. If an IC50 concentration decreases cell viability to 57%, it is expected that with 2X IC50, there should be a sharper fall in cell viability, or is the drug cytostatic?"*

Answer 5: In the first version of the manuscript, the parasite growth experiments had been performed with late-exponential phase parasites. Taking into consideration the reviewer's comment, we repeated the parasite growth experiments (Trypan blue assay, CFSE staining) and we performed these assays with early-exponential phase (log phase) parasites and the relevant results are presented in Figures 2 and 3 of the revised manuscript.

Comment 6: *"In Fig 3A & 3B, Leishmania spp. promastigote proliferation determined by CFSE staining, there are no values stated, just a representative profile."*

Answer 6: We accept the reviewer's comment and in the revised manuscript, all the CFSE relevant data are presented as 2-D line charts expressing the mean fluorescence intensity values ± SD of three independent experiments (Figure 3) and as single parameter histogram overlays representative of one experiment (Figure in S1 Text).

Comment 7: "*For the cell cycle study, it has been stated that 'induced a significant increase in the number of parasites in the sub G0 peak region, consisting the 68.8% ± 8% for IC50 concentration and the 27.9% ± 4.1% 442 for 2 x IC50 concentration, compared to 10.4% ± 5.3% of untreated parasites (negative control group) (Fig 4A, 4B). Why should untreated promastigotes undergo apoptosis? Why should an 2XIC50 conc show a lesser degree of apoptosis than the IC50? The data is very poorly presented."*

Answer 7: Taking into consideration the reviewer's comment, we re-analyzed all the data and the relevant results are presented in Figure 5 and in Figure in S2 Text, in the revised manuscript. Indeed, within the total cell population, the analysis of the percentage of untreated parasites in the sub-G0/G1 region consists of 0.7% and 1.2% of cells for *L. infantum* and *L. major*, respectively, at 24 h.

Comment 8: *"In the Annexin V data, I fail to understand why control cells have such a high % of Annexin V positivity, There are no values stated in the representative profiles."*

Answer 8: Taking into consideration the reviewer's comment, we re-analyzed all the data and the relevant results are presented in Figure 6 and in Figure in S3 Text, in the revised manuscript. The results are plotted as mean values of % annexin V+ parasites ± SD in bar diagrams representative of three independent experiments and as flow cytometric dot plots with respective quadrants, representative of one experiment.

Reviewer No. 2

Comment 1: Results. *"The analysis one are in agreement with the objectives proposed, the results are presented clearly and the figures included in the manuscript are of sufficient quality for clarity. Indeed, some of the FACS images included in the figures can be eliminated for the article publication, keeping just the bar graphics. Such FACS images can be included in a supplementary data file."* **plus Comment 2:** *"As mentioned before, FACS images included in the figures together with the bar graphic are redundant. Such figures can be included in the supplementary data file."*

Answer 1: Taking into consideration the reviewer's comment, the majority of FACS images are included in Supplementary data files, in the revised manuscript. More specifically, single parameter histogram overlays obtained from CFSE assay are presented in Figure in S1 Text. Moreover, single parameter histograms obtained from cell cycle analysis and flow cytometric dot plots obtained from the Annexin-PI assay, for the time-points of 48 and 72 h, are presented in Figures in S2 and S3 Texts, respectively.

Comment 3: *"Regarding experimental infection in mice, data on the swelling of the footpad from infected mice should be included in the manuscript. I know similar results were obtained in previous works already published, but it is interesting to show how the swelling of footpad correlated with those data of parasite burden observed in the lymph node. Further, the authors mention that swelling of the footpad is different in control and TPF treated infected mice, being less severe and without necrosis, a new figure with pictures of the footpad showing such differences in the lesions might be included."*

Answer 3: We accepted the reviewer's comment and thus we inserted Fig 9B in the revised manuscript that illustrates the monitoring of the *L. major*-infection progress in BALB/c mice treated with TPF or HePC or left untreated. As it was correctly mentioned by the reviewer, a new figure presenting pictures with footpad lessions would be an added value to the data of parasite burden in the lymph nodes. Unfortunately, we did not take such photos during the *in vivo* experiment and a repeat of the *in vivo* experiment during the revision period (60 days) was not feasible. This consideration will be seriously taken for our future *in vivo* experimentations.

Editorial Office

Comment of Editorial office: "*We noticed that you used "data not shown" in the manuscript, we do not allow these references. The PLOS data access policy requires that all data be either*

published with the manuscript or made available in a publicly accessible database. Please amend the supplementary material to include the referenced data or remove the references. Please make a note of this change in your response to reviewers."

Answer: "Data not shown" has been removed and all data are within the Revised Manuscript and the Supplementary material.
